# The Institutional Worker

**Published:** 1912-06-08

**Authors:** 


THE
### INSTITUTIONAL WORKER
**Being a Special Supplement to "The Hospital"**

### Editor's Notices.

**Contributions:**

Contributions should be written, or preferably typed, on one side of the paper only, and all articles sent in are accepted upon the distinct understanding that they are forwarded to The Hospital only.

The Editor cannot undertake to return MSS. not used, but when a stamped directed envelope is enclosed the MSS. may be returned if a special request is made.

Accepted articles and paragraphs of news will be paid for after publication at the scale rate.

**Address:**

To prevent delay all contributions and letters on editorial business must be addressed exclusively to the Editor, "The Hospital," 29 Southampton Street, Strand, London, W.C. It is important that this regulation shall be strictly observed.

**Correspondence:**

Correspondence on all subjects is invited. The name and address of correspondents must be given as a guarantee of good faith, but not necessarily for publication.

**Special Articles:**

Special articles are invited, and questions, inquiries, and paragraphs upon all matters relating to the work, administration, and management of general, special, mental, fever, cottage and convalescent hospitals, sanatoria, homes, institutions, societies and organisations for the treatment or care of the sick, injured and dependants of all classes. Every matter affecting the interests and welfare of the staff of all grades working in these institutions will receive special consideration.

**Administrative Medicine, Research and Items of News:**

Special terms will be paid for approved articles on subjects in this wide field by experts who have made some section of it their special study and interest. We wish to give prominence to every movement tending to advance accuracy of diagnosis, efficiency in treatment, and the development of modern methods to eradicate disease and promote the welfare of the suffering.

**A Bureau of Information:**

We invite inquiries and applications for information and help in providing for that numerous class of sufferers who require special care or house-room which they cannot obtain in their own homes or secure for themselves. **We cannot, however, prescribe, or recommend practitioners.**

**Books for Review:**

Publishers are particularly requested to send advance proofs of any new books of importance whenever possible, as the Editor has made arrangements to publish immediate reviews on a new plan.

**Photographs, Plans, Blocks, and Illustrations:**

It is requested that wherever possible MSS. may be accompanied by illustrations in any of the above forms. The name of the sender and of the article to which it belongs should be written on the back of each photograph, drawing, or block for purposes of identification.

**Local Papers:**

Newspapers containing reports or news paragraphs **should be marked** and addressed to the Sub-Editor.

**The Coupon System:**

A coupon will be found at the bottom of the third inside page of the cover each week. This coupon must be attached to every question or inquiry to which an answer is desired in The Hospital.

### Tenders.

The purchase of necessaries in the cheapest market is a factor of the utmost concern in the economical management of an Institution, and one which should engage the serious attention of all officials responsible for the upkeep of any Public Establishment and who desire efficiency in all departments of the Institution under their control.

As it is often impossible locally to secure supplies of the highest quality at the lowest price, special provision is made in our pages for the announcements of Institutions inviting Tenders for Contracts. This, in consequence of the wide distribution of The Hospital, enables Authorities of Institutions to obtain Tenders for articles of any description from practically every quarter of the United Kingdom, and thus ensures that purchases are made at the lowest possible prices consistent with quality.

The charge for notices of Tenders is 6s. per inch, and for this nominal outlay many pounds may be saved in the course of the year. It therefore becomes a sound business proposition for Institution Authorities to announce their requirements periodically in the columns of The Hospital.

**Special Rates** will be quoted for those Institutions that agree to send all their contract, vacancy, and public notices to The Hospital each year, so as to make it a file of such announcements for ready reference.

### Manager's Notices.

**All letters on business matters must be addressed to The Manager, "The Hospital," 28 and 29 Southampton Street, London, W.C.**

**Advertisements:**

To ensure insertion, all advertisements for The Hospital must reach the Manager not later than Wednesday morning in each week. For scale of charges see pages vii and 2.

**Subscriptions,** which may commence at any time, are payable in advance. Cheques and Post-Office Orders should be crossed London County and Westminster Bank, Covent Garden Branch, and made payable to the Manager of The Hospital.

Rates of Subscription (payable in advance).

| | |
|---|---|
| **UNITED KINGDOM.** | |
| Three Months (including Postage) ... ... ... ... | 2s. 0d. |
| Six Months do. ... ... ... ... | 4s. 0d. |
| Twelve Months do. ... ... ... ... | 6s. 6d. |
| **FOREIGN AND COLONIAL.** | |
| Three Months (including Postage) ... ... ... ... | 2s. 6d. |
| Six Months do. ... ... ... ... | 5s. 0d. |
| Twelve Months do. ... ... ... ... | 8s. 8d. |

*SUBSCRIPTION FORM.*

*Please send me* The Hospital *for*..................*months, commencing with your issue of*............................., *for which please find enclosed*.............................

*Name*......................................................

*Address* ......................................................

......................................................

*Date*.......................................

*To* ........................................ *Newsagent.*

**Remittances:**

**It is especially requested that remittances be made by Cheque or Postal Order, and in halfpenny stamps only when the amount is under sixpence.**

### OFFICIAL APPOINTMENTS VACANT.

### Poor-Law Vacancies.

Assistant Lunacy Attendant (£32), Stoke-on-Trent Union. Mr. T. Wood, C. to G., Stoke-on-Trent. June 10.

Assistant Nurses (£25), Pontypridd Union. W. Spickett, C. to G., Union Offices, Pontypridd. June 6.

Barber and Bath Attendant (£30), Stoke-on-Trent Union. Mr. T. Wood, C. to G., Stoke-on-Trent. June 10.

Charge Nurse (£30), Rochford Union. Mr. F. Gregson, C. to G., Southend-on-Sea. June 10.

Charge Nurse (£30), Wolverhampton Union. F. Harrison, C. to G., Poor Law Offices, Wolverhampton. June 10.

Female Attendants (£30), Reading Guardians. Mr. W. H. Oliver, C. to G., Reading. June 12.

Female Assistant Relieving Officer (£80), Leicester Guardians. Mr. H. Mansfield, C. to G., Leicester. June 10.

Female Mental Nurse (£25), Hartlepool Union. Mr. G. Kilvington, C. to G., West Hartlepool. June 11.

Infants' Attendant (£25), Pontypridd Union. W. Spickett, C. to G., Union Offices, Pontypridd. June 6.

Master and Matron (£120 and £60), Stockport Union. Mr. C. F. Johnson, C. to G., Stockport. June 10.

Nurse (£30), Swindon and Highworth Union. Mr. J. P. Kirby, C. to G., Swindon. June 11.

Nurse (£30), Godstone Union. E. A. Head, C. to G., East Grinstead. June 6.

Porter and Portress (married couple) (£40 joint). Sleaford Union. Mr. E. Clements, C. to G., Sleaford. June 3.

Probationer Nurse (£10), Walsall Union. A. H. Lewis, C. to G., 29 Leicester Street, Walsall. June 5.

Probationer Nurses (£10), Croydon Union. Mr. H. List, C. to G., Mayday Road, Thornton Heath, Surrey.

Probationer Nurses (£10), Wolverhampton Union. F. Harrison, C. to G., Poor-Law Offices, Wolverhampton.

Probationers (£10), Birkenhead Union. J. Carter, C. to G., Poor Law Offices, Birkenhead. June 4.

Relieving Officer, etc. (£112, Com. and Fees), Thornbury Union. Mr. H. P. Thurston, C. to G., Thornbury, Glos. June 7.

Relieving Officer, Vaccination Officer, etc. (£60 and fees), St. Asaph Union. Mr. C. Grimsley, C. to G., St. Asaph. June 3.

Relieving Officer (£150), Fulham Guardians. Mr. E. J. Mott, C. to G., 129 Fulham Palace Road, W. June 4.

Relieving Officers (2) (£120), Leeds Union. Mr. J. H. Ford, C. to G., Leeds. June 10.

### Books for Institutional Workers.

| | | |
|---|---|---|
| "Midwife's Pocket Dictionary, with revised Rules of the C.M.B." ... | 1s. | 1d. |
| "The Nurses' Pronouncing Dictionary" ... | 2s. | 0d |
| "A Handbook for Nurses." (J. K. Watson, M.D.) | 5s. | 4d |
| "Art of Feeding the Invalid." ... | 1s. | 6d. |
| "Surgical Ward Work and Nursing." ... | 5s. | 5d |
| "Massage Movements, including the Nauheim Exercises" ... | 1s. | 1d. |
| "Surgical Instruments and Appliances used in Operations" (Harold Burrows, M.B.London, B.S., F.R.C.S.) ... | 1s. | 8d. |
| "The Nurse's Materia Medica" (Herbert French, M.D.) ... | 2s. | 9d. |

Case-papers, Charts, Professional Cards, and every kind of Institutional Literature.

Of all Booksellers or of The Scientific Press, Limited. 28 and 29 Southampton Street, Strand, London, W.C.

